# Prognostic significance of folate metabolism polymorphisms for lung cancer

**DOI:** 10.1038/sj.bjc.6603830

**Published:** 2007-05-29

**Authors:** A Matakidou, R el Galta, M F Rudd, E L Webb, H Bridle, T Eisen, R S Houlston, and the GELCAPS Consortium

**Affiliations:** 1 Section of Cancer Genetics, Institute of Cancer Research, Brookes Lawley Building, Sutton, Surrey SM2 5NG, UK; 2 Department of Oncology, University of Cambridge, Cambridge CB2 2RE, UK

**Keywords:** lung cancer, SNP, prognosis, folate metabolism

## Abstract

Functional nonsynonymous single-nucleotide polymorphisms (nsSNPs) of folate metabolism genes can influence the methylation of tumour suppressor genes, thereby potentially impacting on tumour behaviour. To investigate whether such polymorphisms influence lung cancer survival, we genotyped 14 nsSNPs mapping to methylene-tetrahydrofolate reductase (*MTHFR*), methionine synthase (*MTR*), methionine synthase reductase (*MTRR*); DNA methyltransferase (DNMT2), methylenetetrahydrofolate dehydrogenase (MTHFD1) and methenyltetrahydrofolate synthetase (*MTHFS*) in 619 Caucasian women with incident disease, 465 with non-small cell (NSCLC) and 154 with small cell lung cancer (SCLC). The most significant association detected was with *MTHFS* Thr202Ala, with carriers of variant alleles having a worse prognosis (hazard ratio (HR)=1.49; 95% confidence interval: 1.14–1.94). Associations were also detected between overall survival (OS) in SCLC and homozygosity for *MTHFR* 222Val (HR=1.92; 1.03–3.58) and between OS from NSCLC and *MTRR* 175Leu carrier status (HR=1.36; 1.06–1.75). While there is evidence that variation in the folate metabolism genes may influence prognosis from lung cancer, current data are insufficiently robust to distinguish individual patient outcome.

Lung cancer is a major cause of cancer mortality worldwide. In the United Kingdom, it accounts for more than 33 000 cancer deaths each year (Cancer Research UK). Despite improvements in treatment in recent years, the prognosis from the disease has only marginally improved with 5-year survival rates for both small (SCLC) and non-small cell lung cancer (NSCLC), typically being no better than 15% (Jemal *et al*, 2002). While the major prognostic determinant in lung cancer is stage at presentation, there is variability in survival for patients with same-stage disease. Hence, it is advantageous to identify further prognostic markers, which may aid identification of those patients who will benefit from therapeutic interventions. Furthermore, identifying genes which influence prognosis has the potential to aid the identification of pathways that will be targeted for therapeutic interventions.

Aberrant DNA methylation is recognised as being a common feature of human neoplasia, CpG island hypermethylation and global genomic hypomethylation occurring simultaneously in tumours including lung cancers. Moreover, the cellular profile of DNA hypermethylation has been implicated in progression and metastasis of lung cancer (Nakamura *et al*, 2003; Shimamoto *et al*, 2004).

Variants of folate metabolism pathway (Figure 1) genes such as functional polymorphisms of 5,10-methylene-tetrahydrofolate reductase (MTHFR), affect methylation of DNA and tumour suppressor genes (Kamiya *et al*, 1998; Paz *et al*, 2002), thereby potentially impacting on tumour behaviour. This coupled with the observation that polymorphisms of this pathway can affect the efficacy of cytotoxic drugs (Maring *et al*, 2005) has provided a strong rationale for evaluating such variants as prognostic factors.

Here, we report the impact of polymorphic variation within the folate metabolism pathway genes *MTHFR*, methionine synthase (*MTR*), methionine synthase reductase (*MTRR*), DNA methyltransferase (*DNMT2*), methylenetetrahydrofolate dehydrogenase (*MTHFD1*) and methenyltetrahydrofolate synthetase (*MTHFS*) on lung cancer prognosis in 619 patients. We based our analysis on nonsynonymous single-nucleotide polymorphisms (nsSNPs) as these alter the amino-acid sequence of expressed proteins and are most likely to have functional consequences.

## METHODS

### Patients

Patients with lung cancer were ascertained through the Genetic Lung Cancer Predisposition Study (GELCAPS). Full details about the design and conduct of the study can be obtained elsewhere (Matakidou *et al*, 2005). Briefly, patients were recruited through oncology centres in the UK specializing in the management of lung cancer. To ensure that data and samples were collected from *bona fide* lung cancer cases and avoid issues of bias from survivorship, only incident cases with histologically or cytologically (only if not adenocarcinoma) confirmed primary disease were ascertained. Demographic characteristics (sex, date of birth, ethnic group, area of residence and country of birth, smoking history, history of lung cancer in a first degree relative), treatment and clinical follow-up were collected from cases using standardized questionnaire and proformas. The current analysis is based on 619 female patients all of whom are white Caucasians. Patient characteristics are detailed in Table 1. Ethical approval for the study was obtained from the London Multi-Centre Research Ethics Committee (MREC/98/2/67) in accordance with the tenets of the Declaration of Helsinki. All participants provided informed consent.

### Genotyping

DNA was extracted from EDTA-venous blood samples using a salt extraction procedure and quantified by Picogreen. The 13 nsSNPs genotyped, *DNMT2* Asp112Tyr, *MTHFD1* Lys134Arg, *MTHFD1* Arg653Gln*, MTHFR* Ala222Val, *MTHFR* Arg594Gln, *MTHFR* Thr202Ala, *MTR* Asp919Gly, *MTRR* His595Tyr*, MTRR* Ser175Leu*, MTRR* Lys350Arg*, MTRR* Pro450Arg, *MTRR* Arg415Cys*, MTRR* Ser257Thr. These SNPs were chosen on the basis of being polymorphic in Caucasians, feasibility of genotyping and established functional impact of the sequence changes. Genotyping was conducted by means of Illumina Sentrix Bead Arrays (Illumina, San Diego, CA, USA) according to the manufacturer's protocols (details available on request).

### Statistical methods

Associations between survival and demographic and clinical variables were assessed by means of the *χ*^2^ and Fisher's exact tests. Testing for population substructure was based on examining the distribution of SNP genotypes for evidence of Hardy–Weinberg disequilibrium. Overall survival (OS) of patients was the end point of the analyses. Survival time was calculated from the date of diagnosis of lung cancer to the date of death. Patients who were not deceased were censored at the date of last contact. Median follow-up time was computed among censored observations only. Kaplan–Meier survival curves according to genotype were generated and the homogeneity of the survival curves was tested using the log-rank test. Cox regression analysis (Klein and Moeschberger, 1997) was used to estimate hazard ratios (HRs) and their 95% confidence intervals (CI) while adjusting for radiotherapy and stage. Likelihood ratio testing for the inclusion of covariates and interaction terms was performed to determine the best-fitting model. For each SNP, HRs were generated using common allele homozygotes as the reference group (unless otherwise specified). For polymorphisms with fewer than five minor allele homozygotes, minor allele homozygote genotypes were combined with heterozygotes. In addition, to study the impact of individual SNPs on survival, we evaluated OS as a function of the number of ‘risk alleles’ carried. In this analysis, risk was trichotomised into low, medium and high-risk categories. Owing to the exploratory nature of this study, we reported nominal statistical associations for all analyses. We recognise that examining multiple SNPs risks identification of false associations. However, correction for multiple testing may increase the risk of type II errors (Perneger, 1998). Accordingly, we present uncorrected *P*-values but recognise our exploratory findings require confirmation in another study. This approach minimises loss of true positive results but allows false positive results to be identified (Perneger, 1998; Cuzick, 1999). To adjust for multiple testing, we multiplied *P*-values of each individual test statistic by the number of SNPs in the corresponding gene to obtain a genewide *P*-value, the global *P*-value being the product of the genewide *P*-value and the number of genes. Statistical analyses were undertaken using S-Plus (Version 8, Insightful Corporation, USA). The power to demonstrate a relationship between SNP genotype and OS was estimated using sample size formulae for comparative binomial trials (Farrington and Manning, 1990). In all analyses, a *P*-value of 0.05 was considered statistically significant. To assess the level of linkage disequilibrium (LD) between SNPs, we calculated the pairwise LD measure *r*^2^ between markers mapping to the same gene using the programme PHASE (Stephens *et al*, 2001) that implements the Monte Carlo Markov Chain procedure to estimate two-locus haplotype frequencies. This information was used to investigate the relationship between haplotypes and OS.

### Bioinformatic analysis

We applied two *in silico* algorithms, Polymorphism Phenotyping (PolyPhen) and the Sorting Intolerant from Tolerant to predict the putative impact of missense variants on protein function(Ng and Henikoff, 2002; Ramensky *et al*, 2002). Sorting Intolerant from Tolerant and PolyPhen scores were classified according to the established criteria (Ng and Henikoff, 2002; Xi *et al*, 2004).

## RESULTS

### Study population and SNP genotype distributions

One hundred and fifty-four of the patients (25%) had SCLC, somewhat less than half (43%) presenting with limited disease. Of the 465 patients with NSCLC, 57 (13%) had stage I, 68 (15%) had stage II, 196 (43%) had stage III and 130 (29%) had stage IV disease at diagnosis. The majority of patients with limited stage SCLC had been treated with a combination of radical radiotherapy and chemotherapy, while all patients received chemotherapy (Table 1). The main treatment modality for SCLC patients with extensive disease was chemotherapy. Patients with early stage NSCLC (stage I and II disease) were mainly treated with surgical resection of the primary tumour, while about one-third received chemotherapy and radical radiotherapy. The mainstay treatment modality of patients with stage III and IV NSCLC was chemotherapy. There were 389 (62.8%) deaths in the cohort. Overall the median survival time (MST) was 16.2 months (range 0.03–60.5 months). There were 13 patients with follow-up time less than 1 month, from whom five died. Patients with SCLC had a MST of 17.8 and 11.1 months, if diagnosed with limited and extensive disease, respectively. For NSCLC, by stage, MST ranged from 11.5 months in stage IV patients to 49.2 months in the stage I group. As these survival rates are not significantly different to those documented in audits of lung cancer prognosis (Cancer Research UK), there is no evidence that ‘healthy study participant’ selection will have biased our analyses.

Surgery, any chemotherapy and treatment specifically with platinum-based compounds did not satisfy the proportional hazards assumption required for the Cox model. Therefore, we used a stratified Cox model, stratifying on these covariates. Stage at presentation, histology, radiotherapy, smoking, family history of lung cancer and age at diagnosis were initially included as covariates and backward stepwise selection procedure was conducted to cover the most parsimonious model. Stage and age were included as categorical and continuous variables, respectively. Other factors were coded as binary variables. Factors significantly influencing patient prognosis were stage at presentation (*P*<10^−4^), histology (*P*=0.026) and radiotherapy (*P*=0.0042). Smoking, family history of lung cancer and age at diagnosis did not impact on survival.

### Relationship between SNP genotype and prognosis

For most SNPs genotyped (92%), minor allele frequencies (MAF) were 5% or higher. One SNP was however, observed at comparatively low frequencies (i.e. having MAF <5%). There was no evidence in the data set for population stratification based on testing the distribution of SNP genotypes for Hardy–Weinberg disequilibrium. Thirteen nsSNPs in six genes were assayed. Only SNPs S257T, R415C and P450R, and SNPs H595Y and K175L, all mapping to *MTRR*, were in strong LD (i.e. *r*^2^=1.0 and *r*^2^=0.81, respectively). Hence, the relationship between SNP haplotype and prognosis was restricted to this locus.

There was no correlation between the SNP genotype and pathological parameters, (stage and histology), but in view of the differences in biology of NSCLC and SCLC we also examined for relationships between genotypes and prognosis in the two cell types separately. Table 2 details the relationships between SNP genotype and OS from lung cancer obtained from Cox regression analysis.

**Figure 1:**
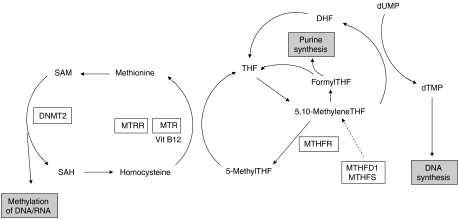
Schematic representation of one-carbon metabolism. MTHFR, methylenetetrahydrofolate reductase; MTR, methionine synthase; MTRR, methionine synthase reductase; DNMT, DNA methyltransferase; MTHFD1, methylenetetrahydrofolate dehydrogenase; MTHFS, methenyltetrahydrofolate synthetase; THF, tetrahydrofolate; DHF, dihydrofolate; dUMP, deoxyuridine monophosphate; dTMP, deoxythymidine monophosphate; SAM, *S*-adenosylmethionine; SAH, *S*-adenosylhomocysteine. Dotted arrow indicates indirect relationship.

**Figure 2:**
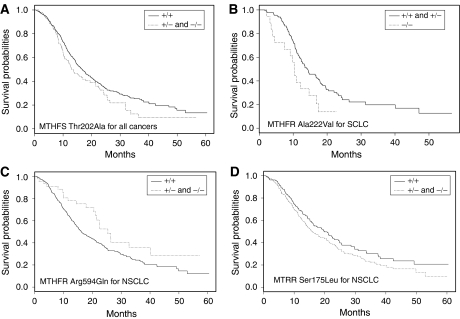
Kaplan–Meier curves for lung cancer patients. (**A**) Relationship between *MTHFS* Thr202Ala genotype and prognosis from all lung cancer; (**B**) relationship between *MTHFR* Ala222Val genotype and prognosis from SCLC; (**C**) relationship between *MTHFR* Ala222Val genotype and prognosis from NSCLC; (**D**) relationship between *MTRR* Ser175Leu genotype and prognosis from NSCLC. The solid line depicts the survival curve for the reference group. +/+, +/− and −/− refer to the common homozygotes, heterozygotes and rare homozygotes, respectively.

Significant associations were identified between polymorphic variation in *MTHFS*, *MTHFR* and *MTRR*. Under the Cox proportional hazards model, the HRs for OS from all lung cancer associated with *MTHFS* Thr202Ala heterozygosity, homozygosity and carrier status were: 1.53 (95% CI: 1.17–2.01), 1.04 (95% CI: 0.38–2.84) and 1.49 (95% CI: 1.14–1.94), respectively. Kaplan–Meier estimates demonstrated that carriers had a shorter MST than patients with the wild-type genotype (MST of 12.9 and 16.7 months, respectively; *P*=0.052; Figure 2). The HRs for OS from NSCLC associated with *MTHFS* Thr202Ala heterozygosity, homozygosity and carrier status were 1.4 (95% CI: 1.02–1.92), 1.45 (95% CI: 0.44–4.71) and 1.4 (95% CI: 1.06–1.91), respectively. The HRs for OS from SCLC associated with *MTHFS* Thr202Ala carrier status was 1.96 (95% CI: 1.17–3.30). For SCLC there were too few homozygotes, hence, these data were pooled with heterozygotes. Variation in *MTHFR* defined by Ala222Val appeared to influence OS for SCLC in a recessive fashion with HR associated with heterozygote, homozygote and carrier status being 1.08 (95% CI: 0.70–1.68), 1.92 (95% CI: 1.03–3.58) and 1.20 (95% CI: 0.79–1.82), respectively. For this group, Kaplan–Meier estimates showed that variant homozygotes had a shorter MST than patients with other genotypes (MSTs of 10.3 and 14.3 months, respectively; *P*=0.025, Figure 2). *MTHFR* Arg594Gln influenced OS with HR associated with carrier status being 0.68 (95% CI: 0.46–1.00), and 0.60 (95% CI: 0.37–0.95) in all lung cancer and NSCLC, respectively. For NSCLC, Kaplan–Meier estimates showed that variant carriers had a longer MST than patients with other genotypes (25.4 and 16.2 months, respectively; *P*=0.02; Figure 1). There was evidence that carrier status for *MTRR* Ser175Leu is associated with a poorer prognosis (HR=1.14; 95% CI: 0.92–1.41) albeit borderline significance in all cancers but significant in NSCLC (HR=1.36; 95% CI: 1.06–1.75). For NSCLC patients, MST in carriers was 15.9 compared to 19.8 months in patients with other genotypes (*P*=0.09, Figure 1). A number of other variants were also associated with OS (Table 2), but none were individually significant.

Evaluating OS as a function of the number of ‘risk alleles’ provided no evidence of an interaction between SNPs (data not shown). Finally, we examined for potential interactive effects between SNPs, response to platinum-based chemotherapy and prognosis. None showed nominally significant interactions at the 5% level.

## DISCUSSION

Major strengths of our study are its large size, the fact that it is population-based, included only patients with incident disease, and has involved the systematic follow-up of patients. We are mindful that it is desirable that studies aimed at identifying prognostic markers should be conducted within the context of a clinical trial to minimise bias. Although bias from non-uniform treatment is a potential confounder in studies of some solid tumours, the management of lung cancer is relatively uniform in the UK, as there are only a restricted number of effective chemotherapeutic agents and prognosis is uniformly poor. Support for this assertion is provided by the fact that survival rates observed in our patient cohort were not different to those expected. It is therefore unlikely that any spurious influences as a consequence of study design will have impacted significantly on our findings. It is well known that the allele frequencies of many SNPs vary among different populations. As our analysis was restricted to white patients, our study findings are unlikely to be confounded by population stratification. The main limitation of our study is the ability to pursue an in-depth examination of the effect of non-genetic factors such as circulating folate levels, which may interact with genotype in defining the clinical behaviour of tumours.

Despite such limitations in this study, we have observed significant evidence for associations between survival and variation in *MTHFR*, *MTHFS* and *MTRR*. Our observation that polymorphic variation in the folate metabolism genes influences cancer prognosis is not without precedent (Alberola *et al*, 2004). We fully acknowledge that we have not captured all variation defined by nsSNPs mapping to all of the folate metabolism genes but our selection was restricted to validated SNPs that could be robustly genotyped using the analytical platform we employed. For example, it would have been desirable to have genotyped nsSNPs mapping to *DNMT1* and *DNMT3b*, given previously published data implicating variation in these genes in development and prognosis of lung cancer (Kassis *et al*, 2006; Kim *et al*, 2006; Wang *et al*, 2006). However, to date only two common (MAF >0.05) validated nsSNPs map to *DNMT1* (Ile311Val and His97Arg) and both unfortunately had low designability for the genotyping platform we employed, thereby precluding evaluation.

We evaluated nsSNPs on the basis that each has the capacity to directly affect the function of expressed proteins, implying a higher probability of being directly causally related to susceptibility. There is good evidence that *MTHFR* Ala222Val directly affect the function of the expressed protein. For SNPs such as *MTHFS* Thr202Ala and *MTRR* Ser175Leu, substitutions are not predicted to be benign. Although such *in silico* predictions about the functional consequences of amino-acid changes are not definitive, these algorithms have been demonstrated in benchmarking studies to successfully categorise 80% of amino-acid substitutions (Savas *et al*, 2004; Xi *et al*, 2004).

The nature of our study precluded us from formally evaluating SNPs in relation to response to radiotherapy as this was only administered to a small number of patients. Similarly only a small number of patients did not receive platinum-based chemotherapy limiting our ability to robustly detect interactions between this type of therapy, genotype and prognosis. Although there may be differences between NSCLC and SCLC, which may reflect differences in biology of the tumour types, our data did not provide real evidence that folate metabolism variation plays a major role in defining differences in prognosis between these tumour types.

In studies of the type we have conducted, there is the issue of adjustment for multiple comparisons. We assessed 13 polymorphisms in seven genes but because more than one polymorphism was tested in some genes, the results are not independent. Hence, for *MTHFS* Thr202Ala, the statistical threshold for global significance is 0.007.

Issues of power are also relevant to the formulation of studies seeking to identify polymorphic variants influencing cancer prognosis. The magnitude of any difference in prognosis associated with individual SNPs is likely to be at best modest hence stipulating significance levels of ∼10^−4^ or less to adjust for multiple testing is inherently unrealistic. For example, for an analysis to have 80% power to demonstrate a 5% difference in survival, which is clinically relevant, would require at least 4800 patient samples to be analysed even if the frequency of the at-risk genotype is 50% stipulating such significance levels. For less frequent genotypes, samples sizes would be impossibly large. On this basis the imposition of very stringent *P*-values (as advocated in genome-wide case–control studies) to outcome studies is questionable creating the serious issue of generating a raft of type II errors (Perneger, 1998).

Despite the strong biologic plausibility and consistency with literature for several individual associations as discussed herein, some of these associations may be false positives as a result of the inherent pitfalls of the candidate gene approach. Hence, individual associations reported in this article must inevitably be interpreted with caution. Nevertheless, even for those true associations, it is unlikely that any individual SNP would have sufficient power to predict clinical outcomes in a disease as complex as cancer. In this context, combined analyses of two or more SNPs in the same pathway are likely to have superior potential to assist in distinguishing different outcome patterns among patients with the same stage disease as even 5–10% differences in prognosis are relevant in a disease. Furthermore, it is plausible that the impact of variation in the folate metabolism genes is likely to be best seen in situations where the pathway plays a major role in defining the efficacy of chemotherapeutic agents in cancers amenable to treatment with agents such as pyrimidine-antagonists (Maring *et al*, 2005).

In conclusion, however attractive the notion that polymorphisms of the folate metabolism pathway genes are in defining cancer prognosis, their role in lung cancer on the basis of our data is minor at best and they are unlikely to have clinical utility.

## Figures and Tables

**Table 1:** Patient demographic and follow-up characteristics

	**No. of patients (%)**
Total	619
Mean age (years)	64.8
	
*Smoking habits*	
Non-smokers	49 (8)
Smokers	570 (92)
	
*Histology*	
SCLC	154 (25)
NSCLC	465 (75)
Squamous	180 (30)
Adenocarcinoma	164 (27)
	
*Tumour stage, by histology*	
SCLC	
Limited	66 (43)
Extensive	86 (57)
	
*NSCLC*	
I	57 (13)
II	68 (15)
III	196 (43)
IV	130 (29)
	
Median survival time (months)	16.2
Events (deaths)	389 (62.8)
Median survival time, months, by histology and stage	
	
*SCLC*	
Limited	17.8
Extensive	11.1
All stages	13.5
	
*NSCLC*	
I	49.2
II	31.9
III	16.2
IV	11.5
All stages	17.6

NSCLC=non-small cell lung cancer; SCLC=small cell lung cancer.

**Table 2:** Relationship between overall survival from lung cancer and polymorphisms in folate metabolism genes

		**All cancer**				**NSCLC**				**SCLC**			
				**Survival**				**Survival**				**Survival**	
**Gene amino acid**	**Genotype**	**Dead (*n*=375)**	**Alive (*n*=219)**	**HR**	**95% CI**	**Dead (*n*=271)**	**Alive (*n*=171)**	**HR**	**95% CI**	**Dead (*n*=104)**	**Alive (*n*=48)**	**HR**	**95% CI**
DNMT2	+/+	259	152		—	188	120		—	71	32		—
Asp112Tyr	+/−	111	64	0.94	0.75–1.19	78	49	0.95	0.72–1.25	33	15	0.97	0.63–1.50
	−/−	5	3	0.75	0.31–1.83	5	2	0.81	0.33–1.98	0	1		—
	+/− and −/−			0.93	0.74–1.17			0.94	0.72–1.23			0.97	0.63–1.50
MTHFD1	+/+	257	146		—	186	114		—	71	32		—
Lys134Arg	+/−	105	64	0.97	0.77–1.22	74	51	1.01	0.76–1.33	31	13	0.83	0.53–1.30
	−/−	13	9	1.46	0.83–2.57	11	6	1.55	0.83–2.87	2	3	1.22	0.28–5.23
	+/− and −/−			1.01	0.81–1.26			1.06	0.81–1.38			0.85	0.55–1.32
MTHFD1	+/+	94	60		—	67	51		—	27	9		—
Arg653Gln	+/−	203	113	0.88	0.68–1.12	153	88	0.96	0.71–1.29	50	25	0.86	0.52–1.40
	−/−	78	46	0.78	0.58–1.07	51	32	0.76	0.52–1.11	27	14	1.2	0.69–2.06
	+/− and −/−			0.85	0.67–1.08			0.9	0.68–1.20			0.96	0.61–1.52
MTHFR	+/+	153	100		—	115	78		—	38	22		—
Ala222Val	+/−	181	100	1.09	0.88–1.36	130	77	1.09	0.84–1.41	51	23	1.08	0.70–1.68
	−/−	41	19	1.37	0.96–1.95	26	16	1.2	0.77–1.86	15	3	1.92	1.03–3.58
	+/− and −/−			1.13	0.91–1.40			1.1	0.86–1.42			1.2	0.79–1.82
MTHFR	+/+	347	192		—	252	148		—	95	44		—
Arg594Gln	+/−	28	26	0.68	0.46–1.00	19	22	0.6	0.37–0.95	9	4	0.95	0.48–1.89
	−/−	0	1		—	0	1		—	0	0		—
	+/− and −/−			0.68	0.46–1.00			0.6	0.37–0.95			0.95	0.48–1.89
MTHFS	+/+	305	182		—	220	142		—	85	40		—
Thr202Ala	+/−	66	33	1.53	1.17–2.01	48	26	1.4	1.02–1.92	18	7	1.96	1.17–3.30
	−/−	4	4	1.04	0.38–2.84	3	3	1.45	0.44–4.71	1	1		—
	+/− and −/−			1.49	1.14–1.94			1.4	1.02–1.91			1.96	1.17–3.30
MTR	+/+	248	138		—	183	104		—	65	34		—
Asp919Gly	+/−	110	70	0.94	0.75–1.19	76	59	0.88	0.67–1.15	34	11	1.1	0.72–1.69
	−/−	17	11	1.44	0.88–2.36	12	8	1.35	0.75–2.45	5	3	1.65	0.65–4.15
	+/− and −/−			0.99	0.80–1.23			0.92	0.71–1.19			1.15	0.77–1.73
MTRR	+/+	302	172		—	219	133		—	83	39		—
His595Tyr	+/−	70	45	1.03	0.79–1.34	51	36	0.99	0.73–1.35	19	9	1.11	0.67–1.82
	−/−	3	2	1.02	0.32–3.25	1	2		—	2	0		—
	+/− and −/−			1.03	0.79–1.34			0.99	0.73–1.35			1.11	0.67–1.82
MTRR	+/+	144	92		—	103	77		—	41	15		—
Ser175Leu	+/−	177	94	1.18	0.94–1.49	125	71	1.42	1.09–1.87	52	23	0.81	0.52–1.26
	−/−	54	33	1.01	0.74–1.39	43	23	1.21	0.85–1.74	11	10	0.62	0.31–1.23
	+/− and −/−			1.14	0.92–1.41			1.36	1.06–1.75			0.77	0.50–1.17
MTRR	+/+	286	164		—	205	126		—	81	38		—
Lys350Arg	+/−	86	51	1.09	0.85–1.39	65	42	1.09	0.82–1.45	21	9	0.91	0.56–1.49
	−/−	3	4	0.79	0.25–2.51	1	3		—	2	1		—
	+/− and −/−			1.07	0.84–1.37			1.09	0.82–1.45			0.91	0.56–1.49
MTRR	+/+	356	210		—	259	164		—	97	46		—
Pro450Arg	+/−	19	9	1.38	0.87–2.20	12	7	1.41	0.78–2.53	7	2	1.32	0.60–2.88
	−/−	0	0		—	0	0		—	0	0		—
	+/− and −/−			1.38	0.87–2.20			1.41	0.78–2.53			1.32	0.60–2.88
MTRR	+/+	356	210		—	259	164		—	97	46		—
Arg415Cys	+/−	19	9	1.38	0.87–2.20	12	7	1.41	0.78–2.53	7	2	1.32	0.60–2.88
	−/−	0	0		—	0	0		—	0	0		—
	+/− and −/−			1.38	0.87–2.20			1.41	0.78–2.53			1.32	0.60–2.88
MTRR	+/+	356	210		—	259	164		—	97	46		—
Ser257Thr	+/−	19	9	1.38	0.87–2.20	12	7	1.41	0.78–2.53	7	2	1.32	0.60–2.88
	−/−	0	0		—	0	0		—	0	0		—
	+/− and −/−			1.38	0.87–2.20			1.41	0.78–2.53			1.32	0.60–2.88

CI=confidence interval; DNMT2=DNA methyltransferase; HR=hazard ratio; MTHFD1=methylenetetrahydrofolate dehydrogenase; MTHFR=methylene-tetrahydrofolate reductase; MTR=methionine synthase; MTRR=methionine synthase reductase; NSCLC=non-small cell lung cancer; SCLC: small cell lung cancer.
